# Stem Cell Therapy for Treatment of Stress Urinary Incontinence: The Current Status and Challenges

**DOI:** 10.1155/2016/7060975

**Published:** 2016-01-10

**Authors:** Shukui Zhou, Kaile Zhang, Anthony Atala, Oula Khoury, Sean V. Murphy, Weixin Zhao, Qiang Fu

**Affiliations:** ^1^Department of Urology, Affiliated Sixth People's Hospital, Shanghai Jiao Tong University, Shanghai, China; ^2^Wake Forest Institute for Regenerative Medicine, Winston Salem, NC, USA

## Abstract

Stress urinary incontinence (SUI) is a common urinary system disease that mostly affects women. Current treatments still do not solve the critical problem of urethral sphincter dysfunction. In recent years, there have been major developments in techniques to obtain, culture, and characterize autologous stem cells as well as many studies describing their applications for the treatment of SUI. In this paper, we review recent publications and clinical trials investigating the applications of several stem cell types as potential treatments for SUI and the underlying challenges of such therapy.

## 1. Introduction

Stress urinary incontinence (SUI) is a common urogenital disease, defined as the involuntary leakage of urine in the absence of a detrusor contraction, generally due to the weakness of the urethral sphincter and pelvic floor [1]. More than 200 million people worldwide suffered from SUI, which seriously affects the quality of life of patients [2]. As a disease whose prevalence is related to advancing age, it affects more women than men with an approximate ratio of 3 : 1 [3]. For women, both pregnancy and vaginal birth are associated with an increased risk of the levator ani muscle defects. Pregnancy and delivery decrease the expression of hypoxia inducible factor-1 and vascular endothelial growth factor [4], which may inhibit the angiogenic response and tissue repair of pelvic floor after childbirth. Likewise, SUI can also affect men and is primarily caused by urethral sphincteric deficiency after radical prostatectomy [5]. At present, several treatments for SUI are available, of which bulking agent injection and Tension-Free Vaginal Tape are the most common and effective therapeutic methods. The various injectable bulking agents applied for the treatment of SUI patients include bovine collagen, carbon beads, silicone, and polyacrylamide hydrogel. However, an ideal periurethral injectable agent for treating SUI has not been found so far. Adverse effects have been reported with all these bulking agents, for example, immunological rejection, sterile abscess formation, foreign-body granuloma, bladder outlet obstruction, and even pulmonary embolism [6, 7]. In addition, due to degradation of bulking agents, their efficacy gradually declines over a period of months or years. Surgery, including sling procedures and bladder neck suspensions, is more efficacious to control the voiding. It is previously reported that the procedures have a 5-year cure rate of more than 80% [8]; however, this procedure has a series of side effects including urinary retention, bladder perforation and hematoma formation. Meanwhile, some patients are not suitable to surgical treatment for contraindication. Most important of all, although the sling procedure and bulking agent injection can enhance the pelvic floor muscles, the urethral sphincter deficiency still remains. Therefore, the key to treating SUI is to improve the mechanism of urethral sphincter insufficiency.

One approach would be the use of stem cells. Stem cells can be easily isolated in high quality and large quantities in vitro and have the potential to develop into any cell type especially during phases of early life and growth. In some organs, stem cells constitute a repair mechanism that is able to replenish cells whenever damage or injury occurs. They are unspecialized cells characterized by a self-renewal property where each daughter cell can either remain undifferentiated or become specialized with a defined function. In addition, provided the appropriate environment and conditions, stem cells can be induced to differentiate into a specific cell-like or tissue-like phenotype with a specifically determined function. Additionally, stem cells are known to have antiapoptotic, antiscarring, and neovascularization effects. Moreover, autologous stem cell transplantation eliminates the risk of immunological rejection. Thus, with their multidifferentiation potential, the stem cells can be induced to differentiate into myoblast to solve the problem of urethral sphincter dysfunction. Here, we summarize relevant progress of stem cell therapy research for SUI and discuss the potential challenges in this paper.

## 2. Stem Cell Transplantation for the Treatment of SUI

**Figure 1:**
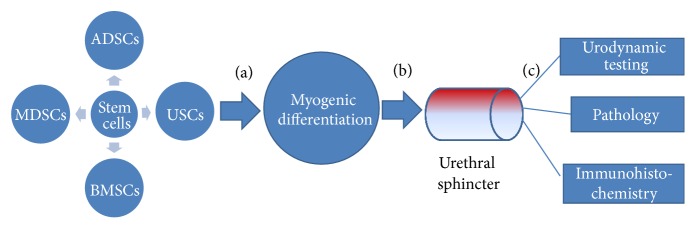
The protocol of stem cell therapy for SUI animal models. In most studies that investigate stem cells as potential treatment for SUI, the following criteria need to be established. (a) The polyphyletic stem cells have plasticity to differentiate into functional cells and proliferate in vitro. Myogenic differentiation is demonstrated with the expression of desmin and a-skeletal muscle actin by immunostaining. (b) They migrate to the damaged external urethral sphincter through periurethral injection. (c) Treatment effects of stem cell transplantation were evaluated through urodynamic testing and morphologic changes of the urethra and frozen urethra sections were submitted to pathology and immunohistochemistry assessment before and after transplantation.

Based on the rapid progress in stem cell biology, stem cells derived from skeletal muscle, adipose tissue, bone marrow, and urine have been used in animal model and preclinical researches for the treatment of SUI in recent years. Most of current papers about stem cell therapy for SUI are focused on animal experiments and follow similar protocols (Figure 1). Furthermore, relevant clinical research was also reported and has showed certain efficacy for the treatment of SUI.

### 2.1. Muscle-Derived Stem Cells (MDSCs) Implantation

Considering that the etiology of SUI is the weakness or dysfunction of urethral sphincter, improving sphincter function may benefit SUI patients. As the name implies, MDSCs are considered to be the predecessor of satellite cells and are not restricted to mesenchymal or myogenic lineages. They have been shown to differentiate into muscle and bone and aid in cartilage healing [9]. Because the source is rich in muscle tissue, MDSCs are easy to obtain in large quantities under local anesthesia. MDSCs are initially isolated from autologous skeletal muscle biopsies and then expanded in vitro and injected into the urethral sphincter. In previous studies, autologous muscle-derived cells have successfully integrated in urethra tissue and partly restored sphincter function in short term [10–13]. However, the potential of muscle-derived cell proliferation is relatively poor, it was often required repeated cell injections to provide enough cells. Furthermore, treatment effect is significantly decreased with time. For the past few years, more studies about MDSCs implantation for treatment of SUI have been reported. Lee et al. [14] reported injection of MDSC into the denervated rats can improve sphincter function, leading to a long term (12 weeks) increase in leak point pressure (LPP) and closing pressure (CP) compared to bovine collagen-injected (LPP: 40.2 ± 7.0 versus 27.8 ± 3.2 cmH_2_O and CP: 30.0 ± 7.6 versus 21.8 ± 1.9 cmH_2_O). Carr et al. [15] reported 1-year follow-up on eight cases of female SUI patients (42–65 years of age) who were treated with MDSCs injections under local anesthesia. Five out of the 8 women (62.5%) showed improvement of SUI symptoms, and one achieved total continence. Besides, Wang et al. [16] showed that inhibiting the fibroblast differentiation of MDSCs induced by TGF-*β*1 could improve the MDSCs-mediated repairing of urethral sphincter function. Xu et al. [17] confirmed the MDSCs-based injection therapies in urethral sphincter restoration can be promoted when combined with biodegradable fibrin glue in a pudendal nerve-transected rat. Compared with MDSCs injection alone, MDSCs plus fibrin glue improved LPP and increased the numbers of surviving MDSCs (109 ± 19 versus 82 ± 11/hpf, *P* = 0.026) and muscle/collagen ratio (0.40 ± 0.02 versus 0.34 ± 0.02, *P* = 0.044) at the injection sites.

However, MDSCs often differentiate quickly without stimulation before they can be implanted and expanded in vivo [18]. For another, the biopsy procedure is painful and requires large muscle biopsies to obtain sufficient MDSCs. Cell Harvesting procedures, if not performed properly, may increase the risk of infection in patients.

### 2.2. Adipose-Derived Stem Cells (ADSCs) Implantation

Currently, ADSCs are the most common stem cell type used in autoplastic transplantation. ADSCs possess more advantages in the clinical application, including sufficient adipose tissue, which can be easily and abundantly obtained by a common surgical procedure and patients have a very high tolerance for repeated sampling. Adipose tissue contains high content of ADSCs, with approximately 15 million ADSCs that can be obtained per gram of adipose tissue and have the ability to proliferate rapidly even in low serum medium. Our group successfully isolated ADSCs from inguinal adipose tissue of rats and induced differentiation of ADSCs into myoblasts with 5-Aza in vitro. Subsequently, the induced ADSCs were injected into the posterior urethral muscularis of rat models with SUI and follow-up analysis showed that ADSCs can be used to treat SUI [19]. Recently, there has been a significant increase in experimental studies about autologous ADSCs transplantation for treatment of SUI. Shi et al. [20] combined ADSCs with silk fibroin microspheres to treat SUI caused by severe intrinsic sphincter deficiency with encouraging results. Injection of silk fibroin microspheres alone only escalated leak point pressures and lumen area in short term (<4 weeks), while the treatment which included ADSCs restored urethral sphincter structure and function in long term (12 weeks after injection). Lin et al. [21] found that implantation of ADSCs through urethral/intravenous injection significantly decreased abnormal voiding rate of SUI rat model compared to control group (33.3% versus 80%). This was accompanied by increased elastin content and smooth muscle content. There was no significant difference in treatment efficacy between the two methods. Kuismanen et al. [22] first described the treatments of autologous ASCs in combination with collagen gel for five female patients with SUI in a pilot study. The mixture of ADSCs and collagen gel was injected transurethrally into the urethral sphincters through a cystoscope. Three out of five patients displayed a negative cough test with full bladder filled with 500 mL of saline and 2 out of 5 patients experienced improvement of symptoms in 12-month follow-up. Zhao et al. [23] used a combination of autologous ADSCs and controlled-release nerve growth factor for treatment of SUI rat by periurethral injection. This treatment enhanced urethral muscle layer distributions, increased the neuronal density of urethra, improved abdominal leak point pressure, and reduced urethral perfusion pressure in SUI rats. Most recently, Silwal Gautam et al. [24] reported that autologous ADSCs injected into cryoinjured rabbit urethras could reconstruct skeletal and smooth muscle areas in the cell-implanted regions. Compared to the cell-free control group, leak point pressure of the cell-implanted group was significantly higher at 14 days after implantation.

ADSCs therapy may have a role not only in the treatment of female SUI, but also in restoring continence in men after radical prostatectomy. Yamamoto et al. [25] provide evidence suggesting that periurethral injection of the autologous ADSCs is a safe and feasible treatment modality for three men (age from 69 to 77 years) with moderate SUI after radical prostatectomy and holmium laser enucleation of the prostate. The bulking effect and increased blood flow were detected at the site of ADSCs injection and persisted during the entire follow-up period (three months), which indicated that the patients experienced excellent short-term outcomes undergoing this cell therapy. However, there are only minimal published studies using stem cell therapy for the treatment of male SUI, and further studies are needed with longer follow-up periods and larger numbers of patients [26]. In brief, the results above indicate the regenerative potential of ADSCs for the treatment of SUI.

### 2.3. Bone Marrow-Derived Mesenchymal Stem Cell (BMSCs) Implantation

BMSCs develop in the bone marrow stromal fraction and are capable of self-renewing and differentiating into several cellular types. They were first described in 1966 as bone forming progenitor cells and have been exploited for 50 years, most recently in tissue engineering. BMSCs are relatively easy to obtain at enough density for therapy. Additionally, they are adherent by nature which makes them easy to grow and expand in culture [27, 28]. As the first stem cells to be described, BMSCs have the capacity to induce urethral sphincter regeneration under special conditions. Data from animal models of SUI showed that injected BMSCs can differentiate into muscle cells and restore resistance of urination. Corcos et al. [29] found that periurethral injection of BMSCs in an animal model of SUI restored the damaged external urethral sphincter and significantly improved valsalva leak point pressure. Gunetti et al. [30] also showed that BMSCs can survive for more than 4 months in absence of immunosuppression and migrated into the muscle among fibers and towards neuromuscular endplates. Kinebuchi et al. [31] transplanted autologous BMSCs into injured rat urethral sphincters to evaluate the functional and histological recovery. Although the leak point pressure outcome showed no significant difference between the BMDSCs and cell-free medium groups during the following 13 weeks, transplanted cells survived and successfully differentiated into skeletal muscle cells, smooth muscle cells and peripheral nerve cells as these were detected by immunohistochemical staining. Kim et al. [32] reported his findings of an animal study which utilized autologous BMSCs to treat female SUI. The cells were cultured to differentiate into muscle lineage cells in vitro and then injected into the denervated external urethral sphincter in female rats. Both, the leak point pressure and the closing pressure recovered at four weeks after the injection of BMSCs, and the injected BMSCs expressed a strong immunoreactivity for muscle-specific markers. Moreover, a recent study confirmed that BMSCs act via their secretome which is collected from cell culture and was shown facilitate recovery of elastic fiber density and pudendal nerve fascicles in this dual muscle and nerve injury SUI model [33]. Nevertheless, similar to MDSCs, the process of autologous bone marrow extraction is painful. Invasive procedures require the collection of many bone marrow samples under anesthesia to obtain enough BMSCs, so it is with an attendant risk of complications.

### 2.4. Urine Derived Stem Cells (USCs) Implantation

USCs can be easily harvested from human voiding urine and expanded in vitro as they do not require enzyme digestion to maintain cell growth. USCs can be repeatedly obtained from urine samples at any patient age, which could easily meet the requirements of cells number and patients are well-tolerated. In addition, it is reported that urine has acute cytotoxicity to damage the survival of the transplanted bone marrow-derived mesenchymal stem cells in vivo [35], while USCs derived from urine have greater ability to resist the toxicity of urine than other stem cell types. Statistics show that up to 75% of fresh USCs can be safely maintained in urine for 24 hours while retaining their original stem cell properties [36]. Thus, USCs may be the most promising cell sources for stem cell-based therapies. Currently, to the best of our knowledge, the experimental study of USCs to treat urinary incontinence is rare. Wu et al. [37] isolated urine derived stem cells from 31 urine samples from 6 healthy individuals with ages ranging from 3 to 27 years. The urine derived stem cells were transfected with an adenoviral vector containing the mouse VEGF gene and injected subcutaneously into athymic mice with a collagen-I gel. The results indicated that more nerve fibers and vascular endothelial growth factor were present in transfected urine derived stem cells and vascular endothelial growth factor overexpression enhanced myogenic differentiation of urine derived stem cells. Liu et al. [38] synthesized microbeads of alginate containing growth factors VEGF, IGF-1, FGF-1, PDGF, HGF, and NGF, which were embedded with USCs in the collagen gel type 1 (2 mg/mL) and injected subcutaneously into nude mice. The growth factors can be released from the microbeads to prolong grafted stem cell survival, promote angiogenesis and nerve regeneration, and stimulate myogenic lineage cell growth in vivo. VEGF-expressing USCs and collagen-I gel injection might indicate the potential to restore continence by muscle regeneration and restoration in the clinic application.

## 3. Tissue-Engineered Suburethral Sling

**Figure 2:**
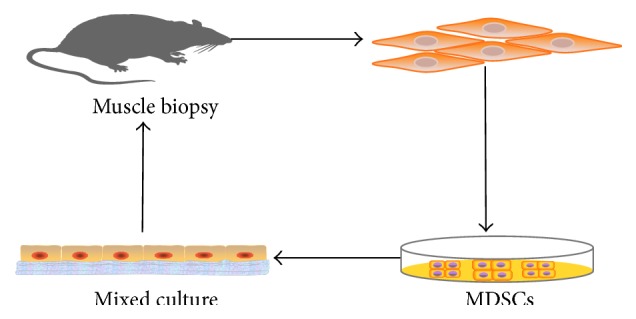
The model of MDSC/SIS Sling therapy for SUI (from [34]). MDSCs were firstly obtained from gastrocnemius muscle and subsequently seeded on a SIS scaffold. The MDSC/SIS Sling was cocultured for 2 weeks in vitro. Finally, the tissue-engineered suburethral sling was placed to repair a damaged urethral sphincter of SUI rat model via a midline transabdominal approach.

Another way to treat SUI is tissue engineered suburethral sling. It is used to support the hyperactive urethra and bladder neck and narrow proximal urethra. It is shown to be an effective therapy for SUI. However, the material of existing suburethral sling lacks ideal compliance and contractility (including excessively lax or stiff). It simply relies on mechanical action to improve urethral resistance. In addition, there may be postimplantation weakening due to scaffold degradation in vivo [39]. Thus, it is difficult to obtain satisfactory curative effect in the long term. The ideal sling material would be safe and durable, would lack antigenicity, and most importantly would be a functional replacement to an impaired continence mechanism. A better effect and fewer complications could be achieved by tissue-engineered suburethral sling made of a degradable material loaded with therapeutic stem cells. Incorporating stem cells into the suburethral sling has obvious advantages, not only to enhance mechanical strength of the sling after implantation but also to promote regeneration of ligament/sphincter surrounding the urethra. Cannon et al. [34] showed that incorporating MDSCs into small intestinal submucosa (SIS) slings does not adversely alter the advantageous mechanical properties of the SIS sling in a rat model of SUI. Implanted MDSCs could migrate into and distribute throughout the SIS and form differentiated myotube structures. Meanwhile, spontaneous contractile activity is observed in MDSCs/SIS constructs by 4 and 8 weeks in culture. MDSCs transplantation reduces the SIS biological resorption in the body and provides the basis for future functional studies of tissue engineered sling materials with long duration (Figure 2). Zou et al. [40] seeded BMSCs into degradable silk scaffolds and implanted them in a SUI rat model. The result showed that both scaffolds with and without BMSCs improved leak point pressure, but only scaffolds with BMSCs led to ligament-like tissue formation over time, which suggested potential long-term function. Most recently, Roman et al. [18] combined ADSCs with thermo-annealed poly-L-lactic acid scaffolds to develop a tissue engineered repairing material. The biodegradable scaffolds with ADSCs produced more key extracellular matrix proteins than oral fibroblasts under the same conditions, which improved the ultimate tensile strength and strain of the scaffolds, thus possessing good application prospect for SUI. Subsequently, Roman Regueros et al. [41] further reported the short-term observation about the acute host response after 7 days of implantation. The result showed good integration into host tissues with extensive host cell penetration (ADSCs), new blood vessel formation, and new collagen deposition throughout the full thickness of the samples. There was obvious difference between cell-containing and cell-free scaffolds. Consequently, surgical replantation of suburethral slings combined with patient's stem cells has great promise to provide better long-term efficiency for SUI patients. However, the “ideal” biomechanical properties require further testing in vivo.

## 4. The Challenges of Stem Cells in the Treatment of Stress Urinary Incontinence

**Figure 3:**
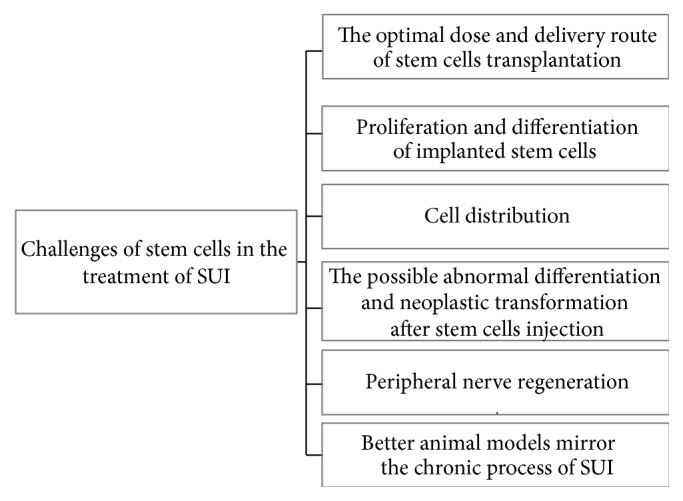
The challenges of stem cells in the treatment of stress urinary incontinence. There are many challenges for stem cells in clinical treatment of SUI, the following facts were shown: (a) an important step to determine the optimal dose and delivery route of stem cells transplantation; (b) how to maintain or promote the ability of proliferation and differentiation of implanted stem cells; (c) the injected stem cells' tendency to gather around the injection site and how to form a uniform cell distribution after stem cell injection; (d) stem cells implantation having the risk of neoplastic transformation and how to avoid the possible abnormal differentiation, even neoplastic transformation, after stem cells injection; (e) peripheral nerve regeneration after stem cell transplantation; (f) better animal models that mirror the chronic process of SUI which should be used in preclinical experiments.

Although stem cell therapy has been reported with encouraging results in the preclinical experiments and had great potential for therapeutic applications of SUI, there are still many challenges in clinical treatment of SUI (Figure 3). The life span of implanted stem cells is relatively short and cell death can be observed within the first week, most probably due to ischemia, inflammation, or apoptosis due to detachment from the extracellular matrix [42]. Meanwhile, the size and function of bioengineered muscle also decline with age [43]. In addition, results of EdU-staining also showed that only a small fraction of the transplanted ADSCs might have differentiated into smooth muscle cells and the majority of the transplanted ADSCs remain undifferentiated [21]. Evidence shows that the inclusion of cytokines and growth factors are able to enhance the ability of stem cell proliferation and differentiation, such as transforming growth factor beta1 [44], vascular endothelial growth factor [45], and basic fibroblast growth factor [46]. Hence, it would be efficient to deliver growth factors to the transplantation site or directly along with stem cells to enhance their differentiation. However, such studies have not been extensively conducted in vivo. Additional studies are needed to further examine the conditions required to better maintain and promote proliferation and differentiation of stem cells after transplantation. One challenge for stem cell therapies is the need for large amount of cells to be transplanted with numbers ranging from 0.5 × 10^6^ to 128 × 10^6^ cells and the appropriate dose of cells is yet to be determined. Current results of stem cell therapy for SUI may not be as reproducible as hoped, the overall success rates (complete continence) ranging from 12 to 79% and improvement rates (quality of life and/or pad test) from 13 to 66% in short-term follow-up [12, 15, 47]. In 3 to 12 months' follow-up, almost all injected cells gathered around the injection site; only a small number of cells were regularly distributed along the normal muscle fiber tissue. Therefore, more studies are needed to achieve a more uniform cell distribution. In the past, experimental results have shown that stem cells transplantation had increased the risk of neoplastic transformation in vivo [48–50]. Moreover, stem cells may promote cancer metastasis through regulating epithelial-mesenchymal transition, including breast cancer [51] and pancreatic cancer [52]. Although the great majority of stem cells have been induced into myogenic cells before urethral injection, how to avoid the possible abnormal differentiation and even malignant transformation in vivo remains unresolved. Additional studies should be carried out for assurance of the long-term safety before stem cells can be used as therapy tools for SUI. Nerve density of urethral tissue declines with age, with a sevenfold age-related loss of nerve density in these same striated urogenital sphincters directly correlated with the loss in striated muscle density in the same tissues [53]. Furthermore, reduced nerve density throughout the striated urogenital sphincter correlates with fewer muscle cells [53]. However, at present, most studies of stem cells therapy for SUI focused on the reconstruction of urethral sphincter and vessel, neglecting peripheral nerve regeneration. More studies are needed to investigate the possible applications of tissue-engineered urethra along with applications to improve distribution of nerve tissue and implanted muscles. Undoubtedly, an ideal animal model for SUI could provide further insight. The formation of SUI is a chronic process that is experienced over years or decades with the urethral sphincter degeneration, while almost all of animal models of SUI mentioned above in the review were established by completely transection of the bilateral pudendal nerve or the vaginal balloon dilatation, which cannot truly simulate the pathophysiology of most of SUI patients. Pauwels et al. [54] established a chronic rat model of SUI with surgical transposition of the urethra to a vertical position. All of the rats leaked urine continuously during the entire study period in the urethral transposition group, while spontaneous recovery of continence (within 1-2 weeks) was seen mostly in the vaginal dilatation group and repetition of the dilatation is needed. Therefore, in order to further comply with clinical practice, more chronic animal models of SUI should be used in preclinical experiments in the future.

## 5. Conclusions

The ultimate goal has always been clinical application of this technology. The published data show that stem cell transplantation as a therapy for SUI has great promise, with some currently used in clinical trials. However, there is still lack of efficient SUI treatments. Additional information is needed from preclinical studies to determine optimal timing and route of stem cell administration. Moreover, many fundamental questions related to the optimal type of stem cells, animal model, implanted stem cells location, stem cells dose, and the long-term safety and efficacy require further examination.
